# Histories of forced sex and health outcomes among Southern African lesbian and bisexual women: a cross-sectional study

**DOI:** 10.1186/s12905-015-0181-6

**Published:** 2015-03-06

**Authors:** Theo Sandfort, M Somjen Frazer, Zethu Matebeni, Vasu Reddy, Ian Southey-Swartz, Southern African Lesbian and Bisexual Women Research Team

**Affiliations:** HIV Center for Clinical and Behavioral Studies, New York State Psychiatric Institute and Columbia University, New York, NY USA; Department of Sociomedical Sciences, Mailman School of Public Health, Columbia University, New York, NY USA; Institute for Humanities in Africa, HUMA, University of Cape Town, Rondebosch, South Africa; Human Sciences Research Council, Human and Social Development Programme, Pretoria, South Africa and University of KwaZulu-Natal, Durban, South Africa; Open Society Initiative for Southern Africa, Johannesburg, South Africa; Division of Gender Sexuality and Health, Columbia University, 1051 Riverside Drive, Unit 15, New York, NY 10032 USA

**Keywords:** Lesbian and bisexual women, Forced sex, Mental health, Southern Africa

## Abstract

**Background:**

Experiences of forced sex have been shown to be prevalent in Southern Africa. Negative outcomes of forced sex have been documented in general populations of women and men and include alcohol abuse, drug use, mental health problems, mental distress, sexual health problems and poor overall health. This study is the first to examine experiences of forced sex and associated health problems among lesbian and bisexual women in Southern Africa.

**Methods:**

This study is based on data collected as part of a collaborative endeavor involving various Southern African community-based organizations. Lesbian and bisexual women in four Southern African countries participated in a cross-sectional survey, for a total study sample of 591.

**Results:**

Nearly one-third of participants had been forced to have sex at some time in their lives. Thirty-one percent of all women reported to have experienced forced sex at least once in their life: 14.9% reported forced sex by men only; 6.6% reported forced sex by women only; 9.6% had had forced sexual experiences with both men and women. Participants experienced forced sex by men as more serious than forced sex by women; forced sex by women was more likely to involve intimate partners compared to forced sex by men. Participants who experienced forced sex by men were more likely to report drug problems, mental distress and lower sense of belonging. Forced sex by women was associated with drinking problems and mental distress. Having experienced forced sex by both men and women was associated with lower sense of belonging to the LGBT community, drug use problem and mental distress.

**Conclusions:**

The findings indicate that forced sex among Southern African women is a serious issue that needs further exploration. Clinicians should be made aware of the prevalence and possible consequences of forced sex among lesbian and bisexual women. Policies and community interventions should be designed to address this problem.

## Background

Researchers have begun to document the prevalence and effects of forced sex among women in Southern Africa [1,2]. However, the specific experiences of lesbian and bisexual women remain unexplored. Given evidence of high rates of sexual violence in these countries [2,3] and human rights reports documenting the violence that targets lesbian and bisexual women specifically [4-7], experiences of forced sex among lesbian and bisexual women are likely to be prevalent. This study is the first to explore psychosocial and health outcomes of forced sex experiences of Southern African lesbian and bisexual women. Based on the same project, we reported earlier on HIV testing and self-reported HIV prevalence in this population [8].

The four countries included in this study (Botswana, Namibia, South Africa and Zimbabwe) belong to the African global burden of disease (GBD) region as defined by the World Health Organization (WHO). According to a report by WHO, 45.6 percent of women in this region have experienced intimate partner violence and/or non-partner sexual violence, compared to 35 percent world-wide [9]. The four countries included in this study vary in prevalence and incidence of rape and other experiences of sexual violence. Population-based samples suggest that Botswana has a lower incidence of rape than South Africa, Zimbabwe and Namibia. For example, one study found a five-year incidence in Zimbabwe of 2.2% compared to just 0.8% in Botswana [10]. Over a quarter of adult men (28%) in a South African representative sample reported perpetrating a rape of a woman [11]. These studies address women’s experiences of forced sex by men; forced sex experiences by women are understudied.

The negative outcomes of forced sex include psychosocial and physical problems. Alcohol abuse, drug use, mental distress, sexual health problems and poor overall health have been found to be associated with forced sex in various studies conducted in the United States [9,12-14]. Little is known about whether consequences of forced sex by women are different from forced sex by men; studies in the United States of intimate partner violence suggest outcomes are similar whether the couple is heterosexual or same-sex [15].

The legal status of same-sex practices and relationships in the countries included in this study varies, with South Africa held up as an example of favorable legal treatment due to the inclusion of protection based on sexual orientation in the country’s constitution [16-18] and Zimbabwe as a model of criminalization [19]. The criminalization of ‘unnatural acts’ in Botswana is often interpreted as to apply to homosexuality [20]. Namibia has some constitutional protections that have been interpreted to mean that sexual minorities should receive similar non-discriminatory treatment; however, this interpretation is not enshrined in law [21].

The variety in the legal situation is however not reflected in the social acceptance of homosexuality. Compared to Western countries, attitudes towards homosexuality in all four countries included in this study are extremely negative. For example, in a representative South African sample, 78% of respondents said that homosexual sex between two consenting adults was ‘always wrong’ [22]. Further, Southern African scholars and activists have documented instances of ‘corrective rape’, in which men force lesbian and bisexual women to have sex to ‘convert’ them into heterosexual women [4-6,22].

In this paper, we use the term forced sex instead of the more common terms ‘rape’ or ‘sexual violence’. We did so for several reasons. First, ‘rape’ has been understood as non-consensual sex in which penetration occurs [2]. Research in South Africa has suggested that discourses of ‘rape’ are often confined by communities to those acts that are committed by “strangers, particularly violent acts, or, gang rape” (p. 1232). Forced sex includes a wider variety of unwanted sexual experiences, including many female same-sex experiences, that do not involve penetration, but that are non-consensual and can have a lasting impact. Unlike ‘sexual violence’, which implies coercion through physical means, ‘forced sex’ can include sex that is unwanted but experienced because of psychological or other forms of non-physical coercion.

As a first exploration we were particularly interested in the diversity of forced sex experiences that lesbian and bisexual women suffer and in the similarities and differences in the consequences to women victims who experienced force sex by men and by women. In terms of potential outcomes we wanted to explore traditional outcomes associated with forced sex including STIs, problems with substance use, and mental distress. In addition, we were interested in exploring whether forced sex experiences might affect women’s sense of belonging. Sense of belonging is seen as a protective factor in people’s health [23,24]. For lesbian and bisexual women it could be that sense of belonging to a sexual minority community and to the community more generally are both of importance for their wellbeing [25,26]. Both could be negatively affected by forced sex experiences. In a context that is not supportive of sexual and gender minority women, the sense of not belonging to the community may be exacerbated by experiences of violence that are not addressed because women are seen to be deserving of these experiences.

## Methods

This study is based on data collected as part of collaboration between university researchers (Matebeni, Reddy, and Sandfort), the Open Society Initiative for Southern Africa (Southey-Swartz and Tallis) and community-based LGBTQ organizations in four countries; Zimbabwe [GALZ (Gays and Lesbians of Zimbabwe)], Botswana [LeGaBiBo (Lesbians, Gays and Bisexuals of Botswana), Namibia (OutRight Namibia), and South Africa (Triangle Project, Durban Lesbian and Gay Community & Health Centre, Forum for the Empowerment of Women, OUT LGBT Well-Being and Behind the Mask).

Research questions, study design and assessment instruments were identified and developed collectively in two one-week meetings and regular conference calls. Staff from community-based organizations collected data after extensive training by the research team in recruitment of participants, data collection, and ethical procedures. Extensive communication by phone took place throughout the implementation of the study. The meaning of study findings and policy implications were explored in a three-day meeting following data collection.

The study was approved by the Research Ethics Committee of the Human Sciences Research Council (South Africa) and the Internal Review Board of the New York State Psychiatric Institute (New York). A waiver of written consent for participation in this study was obtained. Study participation was explained and discussed with participants and each participant was given a study information sheet with contact information of the principal investigator as well as the South African Research Ethics Committee of the Human Sciences Research Council. According to approved procedures, oral consent was subsequently obtained and documented by having the recruiter sign a consent form without naming the individual. A few of the individual organizations involved in participant recruitment compensated participants for their time.

### Procedure

Persons were eligible to participate in the study if their sex assigned at birth was female and if they were 18 years or older; had had sex with a woman in the preceding year; and currently were living in Botswana, Namibia, South Africa, or Zimbabwe. Women were recruited into the study by members of the seven community-based organizations (four in South Africa and one each in Botswana, Namibia, and Zimbabwe; Behind the Mask was not involved in recruitment of participants) that used announcements at relevant meetings and spaces, and gay friendly religious services to advertise the study. Potential participants were furthermore recruited by text messages, cellphone, email and Facebook, making use of organizational data bases. Additional participants were recruited through referral. Data collection took place between September and December, 2010.

Self-administered questionnaires were filled out individually in places that were experienced by the participants as ‘safe’; these places included offices of the community-based organizations, private homes of the participants themselves or their friends, university facilities, cars, or public spaces such as parks and restaurants. Questionnaire completion occurred in the presence of a fieldworker who first explained the purpose of the study and obtained consent. In 15.1% of the cases participants received assistance in filling out the questionnaire. Some women filled out the survey in the presence of other participants, while confidentiality was ensured.

### Participants

In total 591 women participated in the study. With an average age of 26 years, participants in this study were relatively young (Table 1). Most participants identified their race as Black (78.5%), with 12.4% identifying as colored (mixed race; a formal racial classification resulting from the apartheid regime) and the remainder (8.8%) identifying with other population or ethnic groups. Over half of the participants (54.3%) had education higher than secondary school level. The sample was diverse with regards to employment status, with about a third of the women (32.4%) full time employed, 12.4% part time employed, 16.2% students, and 33.7% unemployed. The remaining women (5.2%) had some other employment status (including social assistance). In line with their employment status, over half of all women (51.9%) had no regular income. Over a third of the women (36.1%) received government subsidized health insurance. The majority of the participants reported residing in South Africa (61.6%), with 19.0% reporting living in Namibia, 10.8% in Zimbabwe and 8.6% in Botswana. A small minority of women (8.1%) reported ever having been married. Nearly one quarter (23.7%) had children. Most participants (70.5%) reported attraction only to women, while 29.5% had at least some attraction to both men and women. Most participants (76.9%) identified as lesbian or gay (.7% of the total group identified as gay), while 23.1% of the women identified as some other sexual orientation, including bisexual or heterosexual. Participants averaged close to the middle of five-point scales measuring masculinity (2.57) and femininity (2.93).

**Table 1:** **Characteristics of Southern African lesbian and bisexual women by forced sex experience (N = 591)**

	**Total**	**No forced sex**	**Forced by men only**	**Forced by women only**	**Forced by men and women**	
	**(percent)**	**(n = 407)**	**(n = 88)**	**(n = 39)**	**(n = 57)**	**F/** ***χ*** ^**2**^
Age (mean) (SE)	26.0 (0.27)	25.8	26.5	25.4	26.8	0.73
Race						3.56
Black	464 (78.8)	68.8	14.2	7.3	9.7	
Coloured	73 (12.4)	65.8	17.8	5.5	11.0	
Other	52 (8.8)	73.1	17.3	1.9	7.7	
Education						13.08**
Low	269 (45.7)	73.0	15.7	5.3	6.0^+^	
High	319 (54.3)	64.3	13.8	8.2	13.8^+^	
Employment						24.10*
Full time	192 (32.4)	70.8	16.1	3.1	9.9	
Part time	73 (12.4)	65.8	17.8	4.1	12.3	
Student	96 (16.2)	72.9	14.6	7.3	5.2	
Unemployed	199 (33.7)	67.8	11.6	8.5	12.1	
Other	31 (5.2)	58.1	22.6	19.4^+^	0.0	
Regular income						5.76
No	302 (51.9)	67.5	13.6	8.6	10.3	
Yes	280 (48.1)	70.4	16.8	4.3	8.6	
Health insurance						7.00
No	374 (63.9)	65.5	16.0	7.5	11.0	
Yes	211 (36.1)	75.4	13.3	5.2	6.2	
Country						76.62***
Botswana	51 (8.6)	88.2	11.8	0.0	0.0^+^	
Namibia	112 (19.0)	50.9^+^	9.8	9.8	29.5^+^	
Zimbabwe	64 (10.8)	65.6	17.2	6.3	10.9	
South Africa	364 (61.6)	72.3	16.5	6.6	4.7^+^	
(Ever) married						5.34
No	543 (91.9)	69.8	14.0	6.8	9.4	
Yes	48 (8.1)	58.3	25.0	4.2	12.5	
Has children						27.14***
No	451 (76.3)	72.9	12.2	7.5	7.3	
Yes	140 (23.7)	55.7	23.6^+^	3.6	17.1^+^	
Sexual attraction						5.90
Women only	416 (70.5)	68.3	14.2	8.2	9.4	
Women/men	174 (29.5)	70.1	16.7	2.9	10.3	
Sexual identification						6.14
Lesbian/Gay	452 (76.9)	68.4	14.6	8.0	9.1	
Other	136 (23.1)	70.6	15.4	2.2	11.8	
Gender orientation						
Masculinity, mean (SE)	2.57 (0.05)	2.57	2.44	2.88	2.56	1.10
Femininity, mean (SE)^1^	2.93 (0.05)	2.93^a^	3.25^b^	2.54^a^	2.62^a^	4.04**
General sense of belonging,						
Mean (SE)^1^	2.86 (0.03)	2.91^b^	2.72^a^	2.91^a^	2.67^a^	3.52*
LGBT sense of belonging,						
Mean (SE)^1^	3.25 (0.03)	3.31^c^	3.29^c^	3.11^a^	2.87^b^	10.10***
Drinking problems, mean (SE)^1^	1.71 (0.06)	1.51^c^	1.87^a^	2.78^b^	2.07^b^	10.54***
Drug use problems						
In the last month	207 (35.0)	59.9	19.3	6.8	14.0	4.83**
None in last month	384 (65.0)	73.7	12.5	6.5	7.3	
Mental distress, mean (SE)^1^	2.12 (0.04)	1.97^b^	2.43^a^	2.42^a^	2.53^a^	14.32***
STI symptoms, mean (SE)^1^	1.85 (0.10)	1.55^a^	2.35^b^	2.14^a^	3.44^c^	11.40***

*p < .01; **p < .05; ***p < .001.

^+^Proportions deviating significantly (*p* < .05) from what is expected based on margin totals (standardized adjusted residual > |1.96|).

^1^Means with different subscripts differ significantly (*p* < .05).

### Instrument

Data were collected with an anonymous English-language questionnaire (limited resources prevented translation in local languages). In addition to demographic characteristics, various aspects related to gender and sexuality were assessed in line with recommended practice [27-29]. Sexual attraction was assessed with the question “Do you feel more sexually attracted to women or to men?” Women could choose from the following options: only to women, more to women than to men, to women and men equally, more to men than to women, and only to men. Regarding sexual orientation women were asked: “In terms of your sexual orientation, what do you consider yourself?” Women could choose from the following alternatives: lesbian, bisexual, gay, heterosexual, and other.

Given the fact that the expression of same-sex sexuality in the Southern African context quite often intersects with gender expression [21], we also assessed women’s perception of themselves as masculine and feminine, with two three-item scales [30]. Women were asked to rate themselves on a 5-point scale (Not at all – Extremely) regarding how feminine they perceive themselves, how feminine they act, appear and come across to others, and how feminine their personality is. A parallel scale was used for the assessment of masculinity. Participants were offered the following definitions: “Masculine refers to persons who feel, look and act like ‘real’ men or in a manner which most people think that men should be like. Feminine is the opposite of masculine and refers to what usually is expected from women. People, men or women, who look and behave like ‘real’ women are called feminine”. Cronbach’s alpha for both scales was .91 and .94, respectively.

Experiences of forced sex with men and with women were assessed separately, with a parallel set of questions. Women were asked: “Has a man or boy ever made you have sex when you did not want to by using force or threatening to harm you or someone close to you? This man or boy could have been a stranger, someone you knew, but also your intimate partner”. Women who responded “yes” to the question were asked additional questions about the frequency of these experiences, whether these men/boys were known, whether these experiences ever had happened with an intimate partner, the experienced seriousness of these experiences, as well as what specific kind of sexual experiences were involved. For experiences of forced sex by women, participants answered the parallel set of questions.

To assess women’s sense of belonging to the community in general and to the community of lesbian, gay, bisexual and transgender (LGBT) persons, we adapted scales from Hagerty & Patusky [31] and McLaren [32]. For General Sense of Belonging women were asked to indicate on a 4-point scale (Disagree strongly -- Agree strongly) their agreement with the following statements “Where I live, people accept me”, “I feel misunderstood where I live”, “I am part of the community where I live”, and “I feel like an outsider where I live”. Cronbach’s alpha was .77. For Sense of Belonging to the LGBT community parallel items were asked; Cronbach’s alpha was .77. Drinking problems were assessed with a 4-item scale developed by Mayfield, Mcleod & Hall (Chronbach’s alpha .75) [33]. Women were asked to respond with “yes” or “no” to the following questions: “Have you ever felt you should cut down on your drinking?” “Have people annoyed you by criticizing your drinking?” “Have you ever felt bad or guilty about your drinking?” and “Have you ever had a drink first thing in the morning to steady your nerves or get rid of a hangover (eye-opener)?” Regarding the use of recreational drugs, women were asked if in the preceding 3 months they had used any of the following drugs: marijuana/pot/hash, poppers, cocaine, crack, uppers or speed (amphetamines), downers (barbiturates), psychedelics/LSD/mushrooms/other hallucinogens, heroin/other opiates. Mental distress was assessed with a 10-item short screening scale developed by Kessler and colleagues (Cronbach’s alpha .92) [34]. Women were asked to indicate on a 5-point scale (None of the time -- All of the time) how often they had experienced feelings of distress in the preceding 4 weeks. The scale included feelings such as: feeling tired for no good reason, nervousness, feeling so nervous that nothing could calm you down, feeling hopeless, and feeling that everything was an effort. Finally, women were asked to indicate with “yes” or “no” whether they had experienced nine STI symptoms including strong vaginal odor (e.g., fishy odor); vaginal itching or irritation; light vaginal bleeding (not during monthly bleeding); frequent urination; pain or burning sensation when urinating; lower abdominal pain; thick, cloudy or bloody discharge from the vagina; greenish yellow, possibly frothy vaginal discharge; and pain during sexual intercourse. All yes-answers were added up resulting in a STI symptom index (range 0 to 9).

### Data analysis

To assess differences between participants based on forced sex history (Table 1) and between experiences of forced sex by men and women (Table 2) we used Pearson Chi^2^ tests and one-way analysis of variance as indicated by the level of measurement of the variables involved. Similarly, we used linear regression or logistic regression to assess relationships between forced sex experiences and health outcomes. Missing values were not replaced. All analyses were conducted using SPSS version 22.

**Table 2:** **Characteristics of forced sex experiences (by cases of forced sex (%))** ^**1**^

	**Forced by men**	**Forced by women**	
	**(n = 145)**	**(n = 96)**	***χ*** ^**2**^
Perpetrator was intimate partner	51 (36.4)	55 (59.1)	10.09*
Perpetrator known to participant			3.29
Known	87 (65.9)	68 (77.3)	
Some known and some unknown	15 (11.4)	7 (8.0)	
Unknown	30 (22.7)	13 (14.8)	
Frequency of experience			1.31
Once	61 (46.2)	36 (41.9)	
More than once	71 (53.8)	50 (58.1)	
Type of sex^2^			
Fingers or objects in vagina	55 (50.9)	34 (45.3)	
Fingers or objects in anus	22 (21.0)	16 (23.5)	
Oral sex on participant	34 (32.7)	34 (46.6)	
Oral sex on forcing partner	48 (44.9)	42 (56.0)	
Penis in vagina	108 (85.0)	---	
Penis in anus	34 (31.5)	---	
Stimulated vagina of participant	---	47 (60.3)	
Stimulated vagina of forcing partner	---	40 (54.8)	
Seriousness			29.7*
Not serious at all	32 (22.9)	37 (40.2)	
Somewhat serious	16 (11.4)	25 (27.2)	
Serious	29 (20.7)	17 (18.5)	
Very serious	63 (45.0)	13 (14.1)	

^1^N’s may not sum to total due to missing data; percentages reported are percent of valid data.

^2^Type of sex is percent of total reporting forced sex by this gender; respondents could report more than one type of sex. Differences between proportions not tested.

*p < .001.

## Results

Of the total number of respondents (N = 591), 184 (31.1%) indicated they have had some forced sex experiences some time in their lives; 14.9% of the participants reported forced sex by men only; 6.6% of the participants reported forced sex by women only; 9.6% of the participants reported to have had forced sex experiences with both men and women. Older women were not more likely to have had experiences of forced sex. Participants with a low level of education were more likely to report forced sex with men and women than those with a high level of education (13.8% vs. 6.0%; Table 1). Ever having experienced forced sex was not related to having a regular income or having health insurance. Having had forced sex experiences differed by country; women in Namibia were the most likely to report forced sex experiences (49.1%), especially experiences with both men and women (29.5%). Compared to women in Namibia, women in Botswana and South Africa were less likely to have a history of forced sex experiences with both men and women.

Although there was no association between forced sex and marital status, women with children were more likely than those without to report that they had ever been forced by men (23.6%) or both men and women (17.1%).

Women who compared to other women saw themselves as more feminine were more likely to have had forced sex experiences with men. Sexual attraction (to women only versus to women and men) and sexual identification (lesbian/gay versus other) were not associated with reporting forced sex experiences.

Table 2 compares the participants’ histories of experiences of forced sex by men and by women (women without forced sex experiences do not figure in this Table; participants with forced sex experiences with both men and women are included twice).

In cases of forced sex by women, the forcing partner was significantly more likely to have been an intimate partner compared to cases of forced sex by men (59.1% vs. 36.4%). Whether the perpetrator was known or unknown was not associated with the sex of the forcing person. Sex of the forcing partner was also not associated to the frequency of forced sex experiences: Women who experienced forced sex by men were no more likely to report multiple instances of forced sex than women who experienced forced sex by women.

The most common type of sexual act that occurred when sex was forced by men was vaginal intercourse (85.0%), followed by sex involving putting fingers or objects in the participant’s vagina (50.9%); the most common form of sex that occurred when sex was forced by women was stimulation of the participants’ vagina (60.3%), followed by oral sex on the forcing partner (56.0%). Forced sex experiences by men were more likely to be rated as very serious (45.0%) than forced sex experiences by women (14.1%).

To assess the potential impact of forced sex experiences, we compared participants who reported histories of forced sex by men, by women, and by both men and women with participants who reported no forced sex experiences. Controlling for age, education and country of residence, experiences of forced sex were associated with women’s sense of belonging in general and to the LGBT community, drinking problems, drug use problems, mental distress, and STI symptoms. The associations varied, however, by kind of forced sex experience and outcome (Table 3). Compared to women without forced sex experiences, women who only had forced sex experiences with men were more likely to experience a lower sense of belonging, drinking and drug use problems, and more mental distress, and to report more STI symptoms. Compared to women without forced sex experiences, women who only had forced sex experiences with women were more likely to have drinking problems and to experience more mental distress. Women who had forced sex experiences with both men and women were more likely to experience a lower sense of belonging, in general as well as regarding the LGBT community, drug use problems, and mental distress compared to women without forced sex experiences.

**Table 3:** **Forced sex experiences and health status among Southern African lesbian and bisexual women** ^**1**^

	**Sense of belonging**		**LGBT sense of belonging**		**Drinking problems**		**Drug use problems**		**Mental distress**		**STI symptoms**	
	**β**	***p***	**β**	***p***	**β**	***p***	**AOR**	***p***	**β**	***p***	**β**	***p***
No forced sex	Reference		Reference		Reference		Reference		Reference		Reference	
Forced by men	−0.10	.019	−0.01	.876	0.09	.038	1.92	.008	0.18	<.001	0.14	.002
Forced by women	0.01	.900	−0.07	.116	0.21	<.001	1.27	.497	.011	.007	0.08	.080
Forced by men and women	−0.11	.018	−0.17	<.001	0.07	.116	2.59	.002	.017	<.001	0.23	<.001

^1^Adjusted for age, education and country of residence.

AOR = Adjusted odds ratio.

## Discussion

By documenting these experiences of women we have started to address the information gap about the prevalence, nature and consequences of forced among lesbian and bisexual women. We found that about a third of the Southern African lesbian and bisexual women in our study had experienced forced sex by either men, women, or both. The nature of the forced sex experience was dependent upon the sex of the perpetrator. Forced sex by women was more likely to be perpetrated by intimate partners. Lesbian and bisexual women considered experiences of forced sex by men to be more serious than those by women. Although we have no data to support this, it is possible that men and women use different strategies when they force female partners to have sex; men may be more likely to apply force or cause more damage when they force sex whereas women perpetrators are more likely to use emotional or other non-physical means of force. More work is needed to explore how the role of gender of the perpetrator affects the occurrence of forced sex as well as its consequences.

Our study also provided some evidence of how forced sex affects women’s health and sense of belonging. These associations also differed dependent upon the sex of the forcing partner. General sense of belonging was negatively associated with experiences of forced sex by men but not by women or both sexes. Sense of belonging to the LGBT community seemed to be negatively affected in lesbian and bisexual women who had experienced forced sex by both men and women.

Forced sex by a female partner was strongly associated with drinking problems. In prior studies, lesbian and bisexual women are more likely to have alcohol problems than are heterosexual women, suggesting that this is a familiar method of coping with stressful events [35-38]. While some drinking problems may have pre-dated experiences of forced sex or even contributed to the likelihood of experiencing forced sex, the problem drinking measured in the study was recent; this association needs to be explored in future studies. Drug use was most strongly associated with experiences of forced sex by both women and men. As with drinking problems, drug use may have predated the occurrence of forced sex.

Neither currently available statistics nor this study can shed light on why the rates of forced sex in Namibia are particularly high. These differences could result from how women in the countries were sampled. It is also possible that in different regions there are varying understandings of what constitutes sex and force, partly informed by differences in national or regional differences in sexual scripts.

One finding of particular note is the large percentage of instances of forced sex by men involving penile intercourse (85.0%). It is not clear whether women are more likely to report forced penile intercourse as forced than they are to report other types of forced sex by men. Historically, research has emphasized definitions of rape that involve penile intercourse and further research on other types of forced sex is needed. Legal remedies for forced sex that does not involve penile intercourse may in some cases be limited, and laws and policies related to forced sex must take into account the broad range of sexual practices men and women engage in when they force sex on others.

### Practical implications

The relatively high prevalence of forced sex in this population demonstrates the need for preventive interventions. Such interventions should differentiate between forced sex in intimate lesbian and heterosexual relationships as well as forced sex by other persons than intimate partners. While prevention of sexual violence in general is likely to benefit lesbian and bisexual women, it is unlikely that this will be sufficient. This is particularly so with cases of forced sex that are motivated by the fact that the victim is a member of a sexual minority, such as in corrective rape. Further research is needed to identify what best can be done to prevent forced sex experienced by lesbian and bisexual women in diverse circumstances.

### Future research

Our findings strongly indicate the importance of further research into forced sex experiences of lesbian and bisexual women. To start with, qualitative study of what ‘force’ and ‘seriousness’ might mean to lesbian and bisexual women, how interpretations of what force or sexual approach more generally is are affected by sexual orientation, and how force differs between male and female forcing partners may assist in understanding whether the differences are in degree of physical damage inflicted, feelings of trauma afterward or another issue entirely. Wood, Lambert and Jewkes have noted that in South Africa, a show of force in heterosexual interactions is sometimes used as part of seduction and serves the function of preserving women and girls’ reputations while supporting masculine identity [39]. This suggests that the idea of ‘force’ requires further exploration, particularly in the context of same-sex relationships, in which gendered scripts may (or may not) vary from those proscribed for opposite-sex relationships.

Future research should provide a more in-depth description of the forced sex experience of lesbian and bisexual women, including the age at which forced sex happened, as well as an understanding of the context in which these experiences occur. To what extent are they isolated incidents or part of an ongoing pattern? The data available to us only allowed for a global description. It is not clear to what extent forced sex experiences are associated with physical and emotional abuse. It is not clear whether the forced sex experiences of lesbian and bisexual women with men differ from those of heterosexual women. To better understand the magnitude of the problem it would be informative to compare forced sex experiences of lesbians versus heterosexual women using population-based data.

A related question is when in the process of sexual identity development of lesbian and bisexual women these forced sex experiences take place: is it before or after women start to identify as a member of a sexual minority? Forced sex could also be motivated by the woman being a member of a sexual minority. Such cases have been documented regarding women in South Africa [40,41] and it is likely that such experiences have divergent effects. Our findings also indicate the need of a more in-depth exploration of forced sex between women; is force in relationships uni- or bidirectional? To what extent are such experiences a consequence of how women relate to their same-sex attraction? Could such experiences be prevented by more societal recognition of relationships between women? Creating personal history timelines on which major life events are placed in chronological order might facilitate distinctions between histories of forced sex and additionally indicate whether the negative health outcomes identified here are risk factors, consequences, or mere associations. Longitudinal studies may further help with clarifying this.

Further research to better understand the causes, nature and consequences of forced sex on lesbian and bisexual women will need nuanced methods to assess sexual violence in the lives of lesbian and bisexual women.

### Limitations

As mentioned, due to the cross-sectional study design it is not possible to establish the temporal precedence of women’s experiences of forced sex and their other life circumstances (such as education, employment and income) as well as their current health and psychosocial statuses. This study also did not ascertain in precise detail how often forced sex had occurred and whether it had occurred in childhood or adulthood. While there were two parallel sets of follow-up questions asked about forced sex by men and by women, if women had more than one experience of forced sex by either sex, separate instances of forced sex by people of the same gender could not be differentiated in terms of their seriousness, frequency and character. The age at which forced sex occurred was also not assessed. The study did not address perpetration of forced sex or whether forced sex was reciprocal in nature. A final limitation concerns the nature of the sample: not having the means to do some form of random sampling, we had to rely on non-probability sampling; furthermore, it is not clear how the fact that the questionnaire was only available in English might have affected participation.

## Conclusions

This study suggests that experiences of forced sex are common among lesbian and bisexual women in Southern Africa. The finding that about a third of the women had forced sex experiences suggests the need for greater concern for the safety of lesbian and bisexual women in these countries. Given the criminalization of same-sex sex, negative social attitudes and anti-gay policies of much of Southern Africa, this problem is challenging but necessary for clinicians and policymakers to address.

Forced sex by women was experienced as less severe than that perpetrated by men. This suggests that a restorative approach to prevention of forced sex may be more appropriate and preferable compared to a punitive approach. African use of restorative justice is perhaps best known in post-apartheid (South Africa) and post-genocide (Rwanda) conflict; however, South Africa has also experimented with restorative techniques for serious assaults committed within the context of domestic relationships.

Finally, the associations between forced sex and drinking, drug use, community and LGBT sense of belonging, mental distress and STI symptoms have clinical implications. Clinicians need to be familiar with lesbian and bisexual experiences of forced sex and their associations with negative health outcomes, in order to be able to provide sensitive and adequate care to these populations.
